# Effect of wound fluid on chemotherapy sensitivity of T24 bladder cancer cells with different enhancer of zeste homolog 2 status

**DOI:** 10.18632/oncotarget.18791

**Published:** 2017-06-28

**Authors:** Huan Bi, Zetian Zhang, Li Guo, Cheng Fu

**Affiliations:** ^1^ Department of Urology Surgery, Liaoning Cancer Hospital and Institute, Shenyang 110042, China; ^2^ Department of Technology, Shenyang Yike Biotechnology Co., Ltd, Shenyang 110000, China

**Keywords:** bladder cancer, EZH2, wound fluid, chemotherapy, drug resistance

## Abstract

The present study investigated the effect of zeste homolog 2 (EZH2) and wound fluid (WF) on chemotherapy sensitivities of T24 bladder cancer cells by using a collagen gel droplet embedded culture-drug sensitivity test (CD-DST). T24 bladder cancer cells with different EZH2 expression levels were co-cultured with postoperative WF from patients with bladder cancer. The CD-DST was performed to detect the sensitivity of tumor cells to gemcitabine and cis-diamminedichloridoplatinum (II) (cisplatin, DDP). The survival rates of the bladder cancer cells were used to determine the drug's chemotherapeutic effect. EZH2 knockdown increased the sensitivity of the cells to gemcitabine and DDP, whereas EZH2 overexpression decreased the chemotherapeutic sensitivity. Except for the situation of EZH2 overexpression, co-culturing with WF induced significantly higher drug resistance in tumor cells. Overexpression of EZH2 and surgery-induced WF promoted the drug resistance of bladder cancer cells to the investigated chemotherapeutic agents, suggesting that more studies are needed to investigate the key mechanisms underlying the EZH2- and WF-induced reduction of susceptibility to chemotherapy drugs.

## INTRODUCTION

Bladder cancer is the most common malignant neoplasm of the urinary system, and one of the 10 most common neoplasms in the human body. Bladder cancer has the highest incidence of all genitourinary neoplasms in China [1–3]. Muscle-invasive bladder cancer not only requires radical cystectomy but also adjuvant chemotherapy with the gemcitabine-DDP (GC) regimen [4–7]. The heterogeneity among patients with cancer and tumor tissues causes differences in patient sensitivities to the same chemotherapy regimen, which often increases tumor resistance to chemotherapy drugs [8–11]. The tumor microenvironment also affects the biological behaviors of tumor cells and distal organs, which promotes tumor recurrence and metastasis, thereby causing treatment failure [12–14]. Therefore, identifying potential factors and features of the tumor microenvironment that may affect the effectiveness of chemotherapy not only facilitates the prediction of patient sensitivities to chemotherapy regimens but also serves as a reference for the development of corresponding targeted drugs.

The collagen gel droplet-embedded culture drug sensitivity test (CD-DST) is an *in vitro* anticancer drug sensitivity test used in the chemotherapy of patients with breast and non-small cell lung cancers [15, 16]. This study attempted to use the CD-DST to detect the sensitivities of T24 bladder cancer cells to chemotherapy drugs, which we envisage will lay a foundation for further clinical applications of this technique.

Recently, several studies have suggested that the enhancer of zeste homolog 2 (EZH2) could be a key potential factor in the development of drugs for targeted therapy in multiple tumors, including bladder cancer [17–20]. Furthermore, drugs targeting EZH2 have already entered the research and development phase. Using drugs that have been marketed or developed to treat diseases beyond their indications could save considerable time and economic costs in the research and development of new drugs. For example, bisphosphonates have been used in adjuvant therapy for breast cancer; thereby, enabling the discovery of new uses for old drugs [21]. In this study, we investigated the effects of EZH2 on drug resistance of bladder cancer cells, which could serve as a reference for the future application of inhibitors targeting EZH2 in bladder cancer.

Previous studies have shown repeatedly that post-operative wound fluid (WF) has several biological effects, including the promotion of tumor cell proliferation and drug resistance. Analysis of WF composition has also revealed that certain cytokines (e.g., interleukin [IL]-6) might also play a crucial role in exerting the biological functions of WF [22–23]. Therefore, in this study, the postoperative pelvic WF of patients with muscle-invasive bladder cancer who received a radical cystectomy was incubated with bladder cancer cells with different EZH2 expression levels. This enabled us to observe the concomitant effects of WF and EZH2 on drug resistance of tumor cells. Our results were similar to those of previous studies, but we focused on EZH2, which is a novel and interesting angle from which to explore chemotherapy drug resistance in bladder cancer.

## RESULTS

### Effects of EZH2 on drug resistance of T24 bladder cancer cells

**Figure 1:**
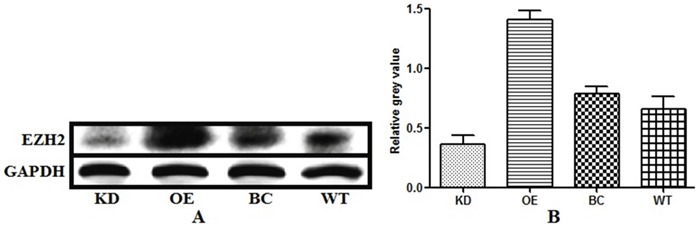
Levels of endogenous enhancer of zeste homolog 2 (EZH2) expression in bladder cancer cells with EZH2 knockdown, EZH2 overexpression, blank vector, and wild-type cells EZH2 expression levels were measured using western blot analysis Glyceraldehyde 3-phosphate dehydrogenase (GAPDH) was the loading control.

**Figure 2:**
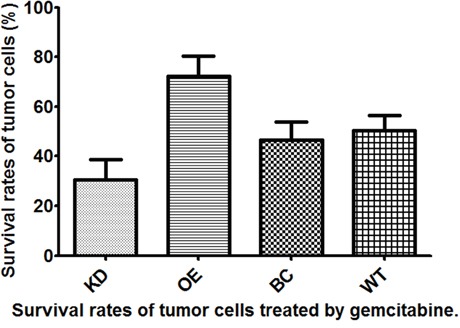
Survival rates of T24 bladder cancer cells from four different subgroups treated with gemcitabine

**Figure 3:**
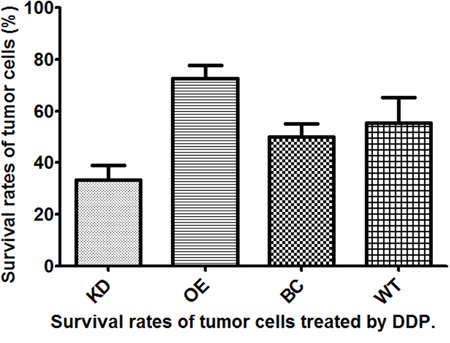
Survival rates of T24 bladder cancer cells from four different subgroups treated with cis-diamminedichloridoplatinum (II) (cisplatin, DDP)

We knocked down and overexpressed EZH2 in parallel groups of T24 bladder cancer cells (Figure 1), which were subsequently treated with the anticancer drugs, gemcitabine and cis-diamminedichloridoplatinum (II) (cisplatin, DDP), survival rates were assessed *in vitro* by performing a CD-DST. The EZH2-knockdown T24 cells showed a remarkable decrease in the survival rate compared to that of the blank vector control and wild-type cells in both gemcitabine and DDP group (p = 0.011 and = 0.002, respectively, for gemcitabine; p = 0.001 and = 0.003, respectively, for DDP). In contrast, the EZH2-overexpressing cells showed a significant increase in the survival rate compared to that of the blank vector control and wild-type cells in both gemcitabine and DDP group (both p = 0.001, for gemcitabine; p < 0.001 and = 0.008, respectively, for DDP) (Figures 2 and 3).

### Effects of WF on drug resistance of bladder cancer cells with different EZH2 status

**Figure 4:**
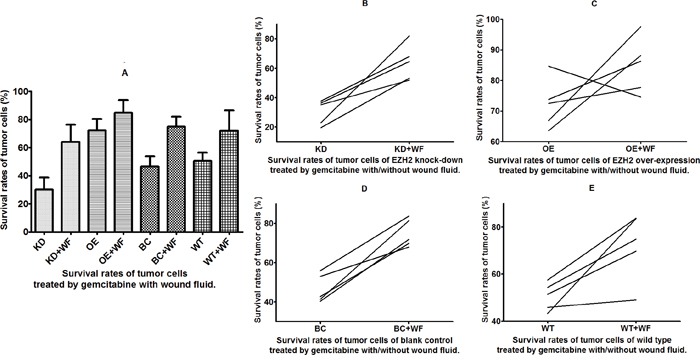
Survival rates of tumor cells treated with gemcitabine and wound fluid (WF) Survival of cells **(A)** treated with gemcitabine with and without WF and **(B-E)** from four different subgroups.

**Figure 5:**
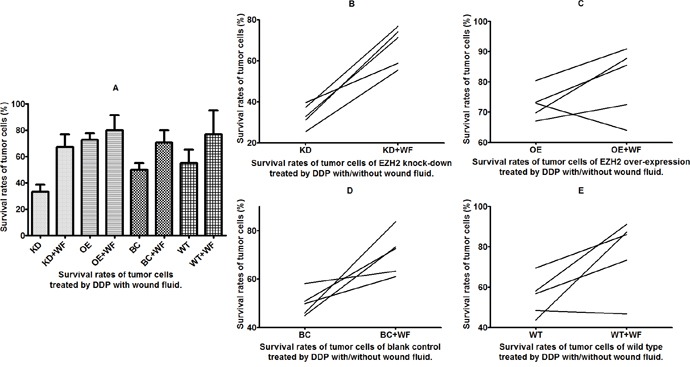
Survival rates of tumor cells treated with cis-diamminedichloridoplatinum (II) (cisplatin, DDP) and wound fluid (WF) Survival of cells **(A)** with and without WF and **(B-E)** from four different subgroups.

The WF-treated cells showed a remarkable increase in survival rates compared with that of the control cells cultured without WF among the different anticancer drug and EZH2 status subgroups (Figures 4A and 5A). The T24 bladder cancer cells with EZH2 knockdown, EZH2 overexpression, and blank vectors, as well as the wild-type cancer cells were cultured with WF, and the survival rates were assessed *in vitro* using the CD-DST after treatment with gemcitabine. The survival rates of cells cultured with WF increased remarkably among the four different EZH2 status subgroups (p = 0.001, 0.049, 0.0003, and 0.013, respectively) (Figure 4B-4E).

The T24 bladder cancer cells with EZH2 knockdown, EZH2 overexpression, and blank vectors, as well as wild-type cancer cells were cultured with WF, and the survival rates were assessed *in vitro* using the CD-DST after treatment with the anticancer drug DDP. The results revealed that the survival rates increased remarkably in cells cultured with WF except EZH2 overexpression subgroup (p = 0.0001, 0.219, 0.002, and 0.047, respectively) (Figure 5B-5E).

## DISCUSSION

There were two main findings in this study. First, changes in EZH2 expression affected the sensitivities of bladder cancer cells to chemotherapy drugs. Specifically, T24 bladder cancer cells with EZH2 knockdown showed increased sensitivities to gemcitabine and DDP in the GC chemotherapy regimen, whereas bladder cancer cells with EZH2 overexpression showed significantly lower sensitivities to these drugs. Secondly, regardless of the EZH2 expression status of T24 bladder cancer cells, incubation with pelvic WF obtained after radical cystectomy in muscle-invasive bladder cancer induced chemotherapy drug resistance in the cells. These results are consistent with the conclusions of previous studies, suggesting that WF promoted tumor cell proliferation, invasion, and other biological behaviors. The results of co-culturing with WF suggests that in agreement with known tumor microenvironment conditions, cytokines in the WF provide a conducive microenvironment for the tumor cells, thereby enhancing their drug resistance [24, 25].

We knocked down and upregulated the expression of EZH2 in parallel groups of bladder cancer cells to compare its biological effects on T24 bladder cancer cells from two opposing perspectives. After transfection with blank vectors, the T24 cells showed a decreased resistance to gemcitabine and DDP, but the difference was not significant. However, after EZH2 knockdown, there was a significant decrease in the drug resistance of tumor cells. In addition, EZH2-overexpressing T24 cells showed a significant increase in drug resistance. Current EZH2-targeting inhibitors have been used clinically to treat ovarian cancer. We demonstrated that EZH2 is a potential target of bladder cancer treatment in basic medical research and identified its clinical implications in patients with bladder cancer.

We used T24 cells in the above experiment as controls and incubated them with WF obtained from patients with bladder cancer in culture medium from the same passaging batch, to detect changes in tumor cell sensitivity to gemcitabine and DDP. The WF from the pelvic drainage tube of patients who were diagnosed intraoperatively with muscle-invasive bladder cancer and received a radical cystectomy. In addition to blood components, the WF mainly consists of tissue fluid components from local tissue damage, which may display certain features of the preoperative tumor microenvironment. Previous studies have shown that the main cytokine in tissue fluid is IL-6, which promotes tumor cell proliferation, invasion, and other biological behaviors via phosphoinositide 3-kinase (PI3K) and other pathways, thereby triggering recurrence and metastasis [22, 26, 27]. Clarifying the postoperative WF components of bladder cancer including the specific component characteristics of patients with different clinicopathological features and providing them with the corresponding management measures, is important. Furthermore, this strategy would enable the in-depth observation of changes in the biological behaviors of tumor cells. This approach could provide the basis for individualized and precise cancer treatments.

In this study, treatment of bladder cancer cells with WF increased their drug resistance. General, the EZH2 knockdown, EZH2 overexpression, blank vector, and wild-type control groups all showed higher drug resistance after WF treatment. The EZH2 knockdown group, which showed a relatively low original drug resistance level, also exhibited the highest increased resistance. After treatment with WF, no significant differences were observed in the drug resistance of the four groups. This indicates that the negative effects of the WF components on tumor cell drug resistance were more significant than the positive effects induced by inhibiting EZH2. Therefore, during chemotherapy and adjuvant therapy targeting EZH2, the effects of WF on local recurrence should also be considered, and the key component factors should be controlled.

This study has a few limitations. Firstly, the WF was from nine bladder cancer patients with tumor invasion of the muscle. Hence, there were no control samples for postoperative WF from tumors at different stages. In addition, the differences in the WF components among the nine patients were not considered, because the samples were all centrifuged, mixed, and divided into 16 equal portions. Therefore, we did not investigate the differential effects of WF from patients with different clinicopathological features on tumor cells. In future studies, the sample size should be increased to establish different control groups. Furthermore, primary tumor cells should be matched with their WF samples to ensure that the experimental conditions simulate real microenvironments in humans as much as possible and, thereby, would produce conclusions that are more reliable.

## MATERIALS AND METHODS

### Cell lines

The T24 bladder cancer cell line used in this study was acquired from Shenyang Yike Biotechnology Co., Ltd., (Shenyang, China).

### WF collection

Nine patients with muscle-involved bladder tumors, who underwent total cystectomies for bladder cancer between July and September 2016 at the Liaoning Cancer Hospital and Institute, China, were enrolled in this study. All the patients had no underlying diseases except for the bladder cancer. Written informed consent was obtained from individual patients, and the experimental protocol was approved by the Ethics Committee of Liaoning Cancer Hospital and Institute.

Drainage WF was collected from the patients on post-surgery day 2. The perforated end of the surgical drain was placed in the wounds until it reached the pelvic cavity. As described previously [high throughput], 50-mL WF samples were collected in sterile containers without additives, centrifuged at 1,600 RCF for 10 min, and then the supernatants were separated into 40 aliquots (2 mL each), which were stored in sterile freezing tubes at −80°C.

### Transfection

The overexpressing EZH2 cells were established using stable transfection. Each vector (1 μg) was transfected into T24 cells using the Lipofectamine Plus kit according to the manufacturer's instructions. Twenty-four hours after the transfection, stable EZH2 clones were selected, and purified bladder cancer cells were transfected with human EZH2-specific or control siRNAs using the Lipofectamine 2000 Reagent (Invitrogen, Carlsbad, CA, USA) according to manufacturer instructions. The EZH2-silenced (EZH2-si) and siRNA-transfected control (EZH2-c) cells were harvested 48 h post-transfection, and the efficacy of the EZH2 silencing was determined using western blot analysis.

### Western blot analysis

The EZH2-sh, EZH2-overexpressing, EZH2-c, and wild-type bladder cells were collected 48 h post-transfection, and the total proteins were extracted using a total protein extraction kit (ProMab, Richmond, CA, USA), followed by centrifugation. The relative levels of targeted proteins to the control (glyceraldehyde 3-phosphate dehydrogenase, GAPDH) were determined using the ImmuNe software.

### CD-DST data acquisition

The CD-DST was performed as previously described by Kobayashi et al. [15, 16]. In brief, bladder cancer cells from each subgroup were washed twice, collected by centrifugation at 250 ×*g* for 3 min, filtered through an 80-um nylon mesh, and then incubated in a collagen gel-coated flask (CG-flask, Nitta Gelatin Inc.) in a CO_2_ incubator at 37°C for 24 h. Only the viable cells that adhered to the collagen gel were collected and suspended in the reconstructed type I collagen solution (Cellmatrix Type CD, Nitta Gelatin Inc.) at a final density of 1 × 10^5^ cells/mL. Three drops of the collagen-cell mixture (30 μL/drop) were placed in each well of a six-well multiplate and a 60-mm dish and allowed to gel at 37°C in a CO_2_ incubator for 1 h. The final concentration was approximately 3 × 10^3^ cells/collagen gel droplet. Culture medium with or without 1% WF was overlaid in each well, and the plate was incubated in a CO_2_ incubator at 37°C overnight.

The anticancer drugs of the GC regimen for bladder cancer chemotherapy tested in the CD-DST were subsequently added (G, 0.5 μg/mL gemcitabine and C, 0.6 μg/mL DDP). The culture time was 24 h for each drug. After the medium containing the anticancer drugs was removed, each well was rinsed twice, overlaid with serum-free culture medium (PCN-1, Nitta Gelatin Inc.), and the plate was incubated for 7 days. On day 4 of the incubation, the medium was changed once. At the end of the incubation, neutral red was added to each well at a final concentration of 50 μg/mL, and the colonies in the collagen gel droplets were stained for 3 h. Collagen droplets in the 60-mm dish were stained just before exposure (day 1). Then, each collagen droplet was fixed with 10% neutral formalin buffer, washed with water, air-dried, and then quantified using image analysis.

### Statistical analysis

A paired *t*-test was used to assess the differences between the two groups while two-sided P-values were calculated for all tests and are reported here. P-values < 0.05 were considered statistically significant. The analyses were performed using the statistical package for the social sciences (SPSS) software (version 19.0, SPSS, Inc., Chicago, IL, USA). The computer program PRISM (version 5, GraphPad Inc., San Diego, CA, USA) was used to create graphs and process the images.

## Abbreviations

WF: wound fluid
CD-DST: collagen gel droplet-embedded culture drug sensitivity test
